# A Comprehensive Analysis of the Blurring Boundary Between Cosmetic Applications and Medical Treatment

**DOI:** 10.1111/jocd.70785

**Published:** 2026-03-19

**Authors:** Eda Kumbasar‐Atay, Kadircan H. Keskinbora

**Affiliations:** ^1^ Private Dermatology Clinic Istanbul Türkiye; ^2^ Department of History of Medicine and Ethics, Faculty of Medicine Bahcesehir University Istanbul Türkiye

**Keywords:** aesthetic medicine, cosmeceuticals, cosmetology, dermatology, patient safety

## Abstract

**Background:**

Dermatology and cosmetology have traditionally been regarded as distinct disciplines, with dermatology focused on the diagnosis and treatment of disease and cosmetology aimed at enhancing aesthetic appearance. In recent years, this distinction has become increasingly blurred due to technological advancements, expanding clinical applications, regulatory gaps, and sociocultural influences.

**Aims:**

This review aims to examine the factors contributing to the blurring boundary between cosmetic and medical dermatologic practices and to evaluate its regulatory, ethical, and patient safety implications.

**Methods:**

A narrative review of peer‐reviewed literature, international regulatory frameworks, and professional guidelines was conducted. Dual‐use dermatologic interventions, including botulinum toxin, dermal fillers, and laser‐ and energy‐based devices, were analyzed across cosmetic and medical indications. Legal approaches in different jurisdictions and sociocultural drivers, such as social media–influenced aesthetic expectations, were also assessed.

**Results:**

The review demonstrates that many dermatologic procedures are positioned simultaneously as cosmetic and medical interventions, creating ambiguity in regulation and professional accountability. The absence of a universally accepted legal definition for “cosmeceuticals,” along with the expanding off‐label use of aesthetic technologies, contributes to inconsistent oversight and potential risks to patient safety. Increased patient demand driven by idealized aesthetic standards further complicates expectation management and clinical decision‐making.

**Conclusions:**

The convergence of cosmetic and medical dermatology highlights the need for clearer legal definitions, strengthened regulatory frameworks, and well‐defined professional standards. A patient safety–centered approach, supported by improved public awareness and professional accountability, is essential to addressing the challenges associated with this blurred boundary.

## 1 Introduction: The Historical and Current Intersection of Cosmetic and Medical Concepts

The history of medicine is a testament to the continuous evolution of its specialties, driven by scientific discovery and societal needs. In this narrative, the distinction between healing and enhancement has long been a foundational principle. It is no longer a simple definitional issue but a challenge to the very structure of healthcare delivery, patient safety, and professional ethics. This article will analyze these forces and their implications, offering a strategic framework for their management.

### 1.1 Definitional Framework: The Distinction Between Cosmetology and Dermatology

Dermatology is a medical specialty focused on the diagnosis, treatment, and management of diseases of the skin, hair, nails, and mucous membranes [1]. The primary aim of dermatology is the promotion and preservation of skin health. This includes the accurate diagnosis and management of dermatological diseases, ranging from common conditions such as acne, eczema, and psoriasis to life‐threatening disorders such as malignant melanoma and cutaneous lymphomas [2]. This discipline primarily aims to heal pathological conditions such as eczema, psoriasis, and acne using medical and surgical methods. Dermatologists also focus on prevention and early detection of skin cancers and other systemic conditions with cutaneous manifestations, recognizing the skin as a window to overall health [3]. Dermatology fundamentally focuses on the concepts of “disease” and “health,” and its main objective is to repair functional disorders and restore the normal physiological state. Dermatology also aims to advance scientific knowledge in areas such as immunology, oncology, infectious diseases, and genetics, as many dermatological conditions overlap with systemic diseases [4]. By integrating clinical practice with research, dermatology contributes significantly to innovation in therapeutics, including biologics, laser therapies, and minimally invasive procedures.

In contrast, cosmetology deals with applications aimed at enhancing the aesthetic appearance, beauty, and overall health of the skin. This field typically includes procedures designed to make the skin look younger, smoother, or more radiant, often in the absence of a disease state. Cosmetology does not deal with diagnosing or treating diseases. Instead, it focuses on appearance improvement and personal grooming. However, it often intersects with dermatology in areas where cosmetic and medical boundaries blur, such as skin rejuvenation procedures, highlighting the importance of professional regulation and safety [5]. Traditionally, cosmetic products and applications cannot claim to have a healing or therapeutic effect. This fundamental distinction has formed the basis of both medical education and legal regulations.

### 1.2 Presentation of the Report's Thesis

The main thesis of this report is that the traditionally clear distinction between dermatology and cosmetology is becoming increasingly blurred under the influence of a multifaceted and complex phenomenon. This blurring not only expands the scope of clinical applications but also has profound effects on legal regulations, marketing strategies, and sociocultural perceptions. The use of related technologies and substances for both aesthetic and medical purposes challenges legal authorities and leads to serious discussions about patient safety, ethical standards, and professional boundaries. This analysis aims to provide a nuanced perspective on the entire issue by detailing this complex transition from legal, clinical, and sociocultural standpoints.

## 2 Methods

This study was designed as a narrative review examining the increasingly blurred boundary between cosmetic applications and medical treatment in dermatology. The review addresses legal, clinical, ethical, and sociocultural dimensions of this phenomenon, with a particular focus on patient safety and professional responsibility. A comprehensive literature search was conducted using PubMed, Scopus, and Google Scholar to identify relevant English‐language publications available up to 2025. Search terms included combinations of cosmetic dermatology, cosmeceuticals, dual‐use technologies, regulatory framework, patient safety, and professional boundaries. Sources were selected based on their relevance to the study objectives and their contribution to understanding regulatory, clinical, and ethical boundaries between cosmetic and medical practice. In addition to peer‐reviewed literature, official legal and regulatory documents were reviewed, including national legislation and guidance from Turkey, the European Union, and the United States.

## 3 Legal and Definitional Framework: “Cosmeceuticals” and Legal Gaps

### 3.1 Definition of the Legal Boundary and Existing Regulations

In Turkey, the cosmetic and pharmaceutical sectors are treated as separate legal categories. The legal framework for cosmetic products is determined by the Cosmetics Law No. 5324, dated March 24, 2005 [6]. Regulations based on this law explicitly prohibit marketing cosmetic products in a way that creates the impression of having a “therapeutic or healing property”. These regulations maintain a clear distinction between a product being a cosmetic or a drug, and they base the system for cosmetic products on notification rather than licensing. This legal distinction aims to protect consumers from misleading health claims. In the United States, products with claims of disease prevention or treatment, such as sunscreens, are considered over‐the‐counter (OTC) drugs and are regulated by the U.S. Food and Drug Administration (FDA) [7]. In the European Union (EU), the key legislation is Regulation (EC) No. 1223/2009, which requires a full technical dossier for each product to be available for inspection by local authorities [8]. Turkey and the European Union were selected for comparison with the United States as they represent different yet internationally influential regulatory approaches. While Turkey is largely harmonized with European Union cosmetic legislation, the European Union emphasizes product safety and post‐market surveillance under Regulation (EC) No. 1223/2009 [8]. In contrast, the United States adopts a predominantly claim‐based distinction between cosmetics and drugs under FDA oversight. Legal gaps arising from ambiguous classification of cosmetic and medical products and procedures, unclear allocation of regulatory authority, and the absence of mandatory medical supervision create fragmented oversight, allowing borderline interventions to be marketed or performed outside standardized medical frameworks and thereby increasing the risk of preventable patient harm [9, 10].

The legal frameworks governing cosmetic and pharmaceutical products in Turkey, the United States, and the European Union all aim to protect consumers but employ different mechanisms. These variations illustrate how cultural, legal, and regulatory priorities shape the categorization and control of cosmetic products in different jurisdictions.

### 3.2 The Legal Key to the Blurring Boundary: The “Cosmeceutical” Concept

The area where the legal framework between cosmetics and treatment is most significantly blurred is with products called “cosmeceuticals.” This term is a portmanteau of “cosmetic” and “pharmaceutical” and refers to cosmetics that contain active ingredients with the potential to positively affect the structure and functions of the skin. Products such as mouthwashes, anti‐dandruff shampoos, sunscreens, and cellulite creams fall into this category. These products may contain biologically derived substances such as vitamins, alpha hydroxy acids (AHAs), herbal extracts, and even growth hormones [11].

However, the legal status of this term is critically ambiguous. While some countries, such as the United States and Japan, accept this product category that lies between cosmetics and drugs, the term “cosmeceutical” is not yet defined in Turkish legal regulations [6]. This definitional gap creates a direct problem with legal oversight. The Cosmetics Law prohibits products from being presented as therapeutic, but cosmeceuticals can claim to “improve” skin functions thanks to their powerful ingredients. Similarly, in the European Union, cosmeceuticals are not defined as a distinct category under Regulation (EC) No. 1223/2009. For example, sunscreens are regulated as cosmetics, categorized as protective products rather than therapeutic ones, which subjects them to less stringent oversight compared to the over‐the‐counter (OTC) drug classification in the United States [7]. Because they lack a legal definition, these products are subject to neither the strict oversight of the drug category nor the simple rules of cosmetics that only promise beautification. To avoid legal penalties, companies often employ ambiguous terminology such as “improves skin structure” or “supports cellular renewal” instead of directly stating therapeutic benefits. While these claims do not overtly violate cosmetic regulations, they create consumer perceptions of medical efficacy, thereby exposing consumers to potential deception [11].

These distinctions, along with the problematic position of cosmeceuticals, can be more clearly understood by comparing the legal definitions and regulatory boundaries across jurisdictions, as outlined in Table 1.

**TABLE 1:** Comparison of legal definitions of the cosmetic categories.^a^

Category	Legal definition (Turkey)	Legal definition (EU)	Legal definition (U.S.)	Important legal boundary (claims, advertising)	Key problem arising from the current situation
Cosmetic products	Substances prepared to be applied to the external parts of the human body	Regulation (EC) No. 1223/2009: Substances intended to be applied to external parts of the human body for cleansing, perfuming, changing appearance, protecting, keeping in good condition, or correcting body odors	Substances for cleansing, beautifying, promoting attractiveness, or altering appearance	Prohibited from giving the impression of having a therapeutic or healing property. In the U.S., products with claims for prevention or treatment of disease are considered drugs	Although the legal definition is clear, marketing practices can indirectly cross this boundary
Drugs	Substances used to treat or prevent diseases	Products presented as having properties for treating or preventing disease, or otherwise affecting physiological functions significantly	Substances intended for use in the diagnosis, cure, mitigation, treatment, or prevention of disease	Clear drug activity and therapeutic purpose	Cosmetic products are subject to strict rules to avoid confusion with drugs
Cosmeceuticals	No legal definition exists	No legal definition exists; products are classified either as cosmetics or medicines depending on claims	No legal definition exists	Technical/functional claims are made on cosmetics, blurring the line with drugs	The legal gap makes oversight difficult and exposes consumers to misleading advertisements

^a^ Data compiled from the Republic of Turkey (2005) (6), Regulation (EC) No. 1223/2009 of the European Parliament and of the Council (8), and U.S. Food and Drug Administration (FDA) guidelines.

## 4 Dual‐Use Technologies and Applications: The Boundary's Reflection in Clinical Practice

The demarcation is most clearly obscured in clinical practice when a single pharmacological agent or medical technology is employed simultaneously for therapeutic and aesthetic indications. Under such circumstances, the identical intervention may be assigned divergent ethical and legal classifications depending on its clinical intent. This duality complicates regulatory oversight and generates critical challenges related to professional responsibility, diagnostic accuracy, advertising ethics, and, above all, patient safety [12]. Cosmetic dermatologic procedures comprise a broad spectrum of interventions that are primarily derived from medical dermatology and performed for aesthetic purposes. These procedures include botulinum toxin injections, dermal filler applications, mesotherapy, platelet‐rich plasma (PRP) and biorevitalization injections; laser‐ and light‐based therapies (including treatment of pigmentary and vascular lesions, laser hair removal, tattoo removal, fractional laser applications, and intense pulsed light [IPL]); as well as chemical peels, microneedling, radiofrequency‐ and high‐intensity focused ultrasound–based skin rejuvenation procedures [13].

### 4.1 Botulinum Toxin: A Perfect Example of Aesthetic and Medical Use

Botulinum toxin type A is a neurotoxin produced by *Clostridium botulinum* . Although widely recognized for its cosmetic application in reducing facial wrinkles, botulinum toxin has a long‐standing role in clinical medicine. By inhibiting the release of acetylcholine at the neuromuscular junction, it induces temporary muscle relaxation, making it useful in treating a range of neurological, ophthalmological, dermatological, and urological conditions [14].

Botulinum toxin is widely used in the aesthetic field to smooth dynamic wrinkles caused by the movement of facial muscles. Forehead, glabella, and periorbital wrinkles are among the main targets of this application. It is also preferred for aesthetic purposes, such as eyebrow lifting and a gummy smile.

Botulinum toxin represents one of the most frequently performed minimally invasive aesthetic interventions worldwide, consistently ranking at the top of global procedure statistics [15].

However, the use of botulinum toxin is not limited to aesthetic purposes; the same substance is also used in the treatment of various medical conditions. These include the prevention of chronic migraines, the reduction of excessive sweating (hyperhidrosis), bruxism (grinding of the teeth), and the control of muscle spasms in neurological diseases such as cerebral palsy, cervical dystonia, spasticity, and blepharospasm [16].

The use of the same substance as an aesthetic “service” (improving appearance) and a functional “treatment” (relieving pain or correcting muscle dysfunction) raises complex legal and ethical questions. Therapeutic indications of Botulinum toxin, such as chronic migraine or cervical dystonia, are recognized as medical treatments and are therefore eligible for reimbursement under public or private insurance [16]. On the other hand, wrinkle treatment with the same substance is typically considered “cosmetic”. Unnecessary or excessive applications are often driven by “demand” rather than the principle of “medical necessity,” which raises ethical issues regarding the use of a drug without a clear medical indication. This shows that the purpose of the application becomes more important than the application itself.

### 4.2 Dermal Fillers: From Reconstruction to Aesthetic Enhancement

Dermal fillers are defined as medical devices intended for injection into the dermis or subcutaneous tissue to restore volume, correct wrinkles and folds, or enhance facial contours [17]. They are typically gel‐like substances with varying degrees of viscosity and longevity, depending on their chemical composition [18].

In the cosmetic field, dermal fillers are used to restore volume loss, smooth wrinkles, and shape lips, cheekbones, or the jawline. These applications generally aim to achieve a youthful, fresh, and plump appearance. The most common types of dermal fillers: Hyaluronic Acid (HA) Fillers, Calcium Hydroxylapatite (CaHA), Poly‐L‐lactic Acid (PLLA), Polymethylmethacrylate (PMMA) [19].

Dermal fillers have the ability to restore lost volume, correct contour irregularities, and stimulate tissue remodeling, making them valuable tools in reconstructive and therapeutic contexts. Recognizing their dual role is essential for understanding both their clinical significance and their regulatory categorization as Class III medical devices [20].

Dermal fillers can also be used in the treatment of pathological conditions such as soft tissue defects resulting from trauma, burns, or surgical resections. They can correct asymmetry, scarring, or localized tissue loss, serving as minimally invasive alternatives or complements to surgical reconstruction [21]. Fillers may be used to correct congenital craniofacial abnormalities, such as hemifacial microsomia or lip deformities [22].

In cosmetology, dermal fillers occupy a central position among minimally invasive procedures. Unlike their therapeutic role in reconstructive medicine, cosmetic applications are primarily focused on restoring youthful appearance, enhancing aesthetic features, and improving skin quality. The cosmetic indications of dermal fillers are correction of wrinkles and folds, lip augmentation, periorbital rejuvenation, nonsurgical facial contouring, hand rejuvenation, and scar revision [23]. With the expanding use of dermal fillers, an increase in reported complications is anticipated [24]. Importantly, various complications may occur even in the hands of experienced injectors. Complications associated with dermal filler injections may be categorized as early‐onset or delayed‐onset based on the timing of clinical presentation. Early‐onset complications, occurring within hours to days after injection, commonly include injection‐site reactions such as edema, erythema, pain, ecchymosis, hypersensitivity reactions, infection, and vascular occlusion, which may result in local tissue necrosis or embolic events. Delayed‐onset adverse effects, developing weeks to years after treatment, include biofilm‐related complications, foreign‐body granuloma formation, dyspigmentation, and scarring. Although many reactions are transient, severe complications including intravascular embolization leading to blindness or stroke highlight that dermal filler procedures are invasive medical interventions requiring appropriate clinical expertise and patient safety oversight [25].

### 4.3 Laser and Light Therapies: Disease and Beauty Goals

Laser and light‐based therapies are extensively employed in cosmetic dermatology, particularly in procedures aimed at skin rejuvenation, the management of pigmentation disorders, and laser‐assisted hair removal [26]. These technologies aim to make the skin surface smoother and more uniform. Ablative lasers (e.g., CO_2_ or Er: YAG) vaporize the outer layers of skin to treat severe wrinkling, seborrheic keratosis, warts, and atrophic scars, while non‐ablative lasers act by inducing controlled dermal injury while preserving the epidermal barrier. This mechanism stimulates collagen remodeling, neovascularization, and tissue regeneration, leading to clinical improvement with reduced downtime and lower complication risks [27].

However, laser and light therapies are also used in the treatment of specific pathological conditions, such as vascular malformations, such as port‐wine stains, hemangiomas, telangiectasias, rosacea, leg varicose veins, and spider veins [28]. In the field of oncology, lasers play a significant role in the treatment of superficial basal cell carcinoma and actinic keratoses, often in combination with photodynamic therapy [29]. These conditions are considered dermatological diseases or functional disorders beyond being merely an aesthetic issue. Laser and light therapies have revolutionized dermatology by offering controllable and selective treatment for a wide variety of conditions, from vascular lesions to scar revision and tattoo removal. The clinical success of laser treatments depends on a complex interplay of patient‐related, device‐related, and operator‐related factors, all of which influence both safety and efficacy [30]. From a patient‐related perspective, the most critical determinant is skin type, commonly classified according to the Fitzpatrick phototype scale. Individuals with darker skin (types IV–VI) are at increased risk of post‐inflammatory hyperpigmentation, hypopigmentation, or scarring following laser treatment, requiring careful selection of wavelengths and energy parameters [31]. On the other hand, the outcome depends on the type of laser and its parameters, including wavelength, fluence (energy per unit area), pulse duration, and spot size. Advances in technology, such as fractional photothermolysis and cooling systems, have significantly improved both safety and patient comfort [32].

The complexities described above are not merely theoretical but manifest in tangible clinical practices. In particular, interventions such as botulinum toxin injections, dermal fillers, and laser/light–based devices demonstrate how a single technology can simultaneously serve aesthetic and therapeutic goals, thereby complicating legal definitions, reimbursement pathways, practitioner competencies, and informed consent. These ethical and clinical challenges become more apparent when the interventions are reviewed side‐by‐side, as synthesized in Table 2.

**TABLE 2:** Therapeutic and cosmetic interventions with ethical/clinical considerations.^a^

Technology/Substance	Cosmetic use cases	Medical/Therapeutic use cases	Ethical/Clinical challenges posed by this duality
Botulinum toxin	Wrinkle reduction, eyebrow lifting, gummy smile, and facial contouring	Chronic migraine treatment, excessive sweating, bruxism, and neurological disorders	The same substance changes legal and insurance status based on its purpose. It creates a purpose‐driven distinction between treatment and aesthetics
Dermal fillers	Restoring facial volume loss, wrinkle filling, lip, chin, and cheekbone augmentation	Filling atrophic acne scars, correcting traumatic or postsurgical scars, and correcting facial symmetry disorders	This leads to the perception of “beautification” as a “treatment,” which complicates patient expectations and informed consent processes
Laser and light therapies	Skin rejuvenation, hyperpigmentation treatment, hair removal, tattoo removal, striae treatment, acne scars	Vascular lesions (hemangioma, rosacea, varicose veins), spider veins, scar revision, tinea unguium, and wart treatment	Technology itself can be used in both cosmetic and medical fields, depending on the purpose of use. This creates uncertainties about practitioner competence and patient safety

^a^ Data compiled from King et al. [33], Cassalia et al. [34], and Qureshi et al. [35].

## 5 Sociocultural Dynamics and Market Forces: Social Factors Behind the Blurring Boundary

### 5.1 The Role of Social Media: The Obsession With Perfection and Virtual Beauty Perception

One of the most important reasons for the increase in interest in cosmetic procedures is the widespread use of social media. According to plastic surgeons, social media has triggered the need for aesthetic procedures and a beauty obsession. A recent systematic review demonstrated that social media exerts a significant influence on individuals' decisions to undergo cosmetic procedures, shaping both their aesthetic perceptions and treatment‐seeking behaviors [36].

Platforms such as Instagram and TikTok have played a pivotal role in popularizing digital filters that can instantly alter facial features, thereby fostering unrealistic aesthetic expectations. This continuous exposure to “flawless” images can lead to increased body dissatisfaction, particularly among adolescents [37]. This persistent exposure fosters low self‐esteem and fear of negative evaluation, all of which are strongly associated with generalized anxiety disorder and social anxiety disorder [38].

Aesthetic operations, which used to be a hidden matter, have become a matter of prestige thanks to social media. This situation creates a market dynamic where a medical procedure is marketed as a “lifestyle” product that can be showcased on social media, rather than a “need.” This blurring is not just a clinical and legal phenomenon but also a sociocultural one where society's perception of beauty and the body challenges the boundaries of medicine [39].

Due to the pressure exerted by social media, there has been a growing demand for cosmetic procedures, particularly among adolescents. Consequently, significant dermatological conditions are often mismanaged by unqualified individuals, leading to a serious public health concern.

### 5.2 The Evolution of the Beauty Industry and the “Medical Aesthetics” Concept

Traditional beauty centers have experienced a shift towards the medical field by adding semi‐invasive procedures such as Botulinum toxin, dermal filler injections, and laser therapies to their service portfolios under the name “medical aesthetics”. This has led to beauty centers offering “treatment” services and the erosion of the boundaries between areas of expertise. This shift reflects the commercialization of medical technologies within non‐clinical settings and raises important concerns regarding professional oversight, patient safety, and regulatory accountability [40]. While plastic surgeons have a broader scope that includes both reconstructive and aesthetic surgery, beauty salons and medical aesthetics clinics are pushing these professional boundaries. Consumers find it difficult to distinguish between the quality of the places offering these services, and there is a risk of unqualified individuals performing procedures beyond their authority. This is a significant problem that endangers patient safety.

## 6 Ethical and Professional Challenges: Critical Issues Arising From the Blurring Boundary

### 6.1 Patient Safety and Informed Consent

Every injection and laser application, whether for cosmetic or medical purposes, has side effects that can be mild and temporary or, in rare cases, permanent. For example, a temporary headache, swelling, or bruising may occur after a Botulinum toxin application. Dermal filler applications may also lead to mild bruising, tenderness, and swelling, but serious complications like vascular occlusion can result in tissue necrosis, scarring, or, in extremely rare cases, blindness if filler material obstructs the retinal artery [33].

One of the critical considerations for patient safety in cosmetic interventions is the obligation that substances administered through intradermal injection be categorized under Class III medical devices. In procedures such as mesotherapy, which are widely performed in cosmetic practice, certain pharmaceutical products are administered intradermally despite being formally approved only for topical application. Agents formulated for topical use may contain preservatives, stabilizers, or excipients that are biocompatible on the skin surface but potentially harmful if introduced into vascular or connective tissues. This creates a risk of local tissue necrosis, granulomatous inflammation, or embolization, as well as delayed hypersensitivity reactions [33].

This off‐label use raises significant concerns regarding patient safety, as the pharmacokinetics, systemic absorption, and potential adverse reactions of such agents have not been adequately studied for injectable administration.

However, the perfect obsession created by social media can cause patients to seek these procedures with unrealistic expectations. This complicates the informed consent process. Patients are more prone to overlook potential risks because they are focused on the idealized results seen on social media. This places an additional ethical responsibility on physicians to inform the patient not only about the mechanical details of the procedure but also about the risks and realistic expectations [33]. Social media has been linked to a higher prevalence of psychological conditions such as body dysmorphic disorder (BDD) among patients seeking cosmetic procedures [41].

### 6.2 Professional Competence and the Need for Interdisciplinary Collaboration

Plastic surgery is a broad field that encompasses reconstructive and aesthetic surgery. Similarly, dermatology offers a wide range of services, from the treatment of skin diseases to cosmetic applications. This multi‐disciplinary nature creates issues of authority and responsibility regarding who should perform which procedures. For example, botulinum toxin can be used in different specialties for a variety of conditions, from neurology for migraines to dentistry for teeth grinding [42].

This situation creates a conflict in terms of professional boundaries and responsibilities. The multi‐disciplinary nature of the applications necessitates greater cooperation among professional medical organizations and the establishment of common training standards. The blurring of boundaries makes it difficult for patients to choose the right specialist and fuels debates about competence and ethical responsibility among healthcare professionals.

Accurate diagnosis and treatment of dermatological conditions should be carried out by dermatologists, as this is essential for ensuring patient safety. Malignant melanoma, for instance, is a potentially life‐threatening disease that may be misinterpreted as a benign pigmented lesion by non‐specialists, such as laser operators. Such misdiagnoses can result in inappropriate interventions, delays in proper treatment, and consequently, serious or even fatal outcomes [43].

## 7 Discussion

The multidisciplinary nature of aesthetic applications creates a significant conflict regarding professional boundaries and responsibilities. With a variety of specialists, from plastic surgeons and dermatologists to non‐physician practitioners, performing similar procedures, the questions of who should perform what and under what authority become paramount.

A critical dimension of this debate is patient safety. The accurate diagnosis and treatment of dermatological conditions are essential to ensuring patient safety and must be carried out by a dermatologist. A malignant melanoma, a potentially life‐threatening disease, can be misinterpreted as a benign pigmented lesion by non‐specialists, such as laser operators. Such a misdiagnosis can lead to inappropriate interventions and delays in proper treatment, resulting in serious or even fatal outcomes [44]. Studies have shown that misdiagnoses of melanoma, particularly on the feet, can lead to a significant delay in diagnosis, which is associated with a poorer prognosis and a lower five‐year survival rate [34].

The convergence between cosmetic applications, surgical and medical treatment is a complex strategic issue that demands a sophisticated, multi‐pronged response. By strengthening legal frameworks, enhancing public education, and clarifying professional standards, this evolving landscape can be navigated in a manner that safeguards patient safety and preserves the integrity of medical practice [45].

The distinction between cosmetic and medical treatment has evolved into a complex spectrum that cannot be defined by a single line and is in constant flux. This blurring has emerged from a combination of technological advancements (e.g., lasers and Botulinum toxin), legal gaps (e.g., the concept of “cosmeceuticals”), and sociocultural dynamics (e.g., the influence of social media). This situation reveals the reality that many procedures labeled “cosmetics” are, in fact, medical procedures performed for non‐therapeutic purposes. This phenomenon presents new challenges in terms of patient safety, professional ethics, and legal oversight that require urgent solutions [35].

Future recommendations must therefore be developed to provide a structured response to these ethical, professional, and regulatory challenges. To manage this complex problem area and mitigate future potential risks, the adoption of the following multi‐faceted approach is recommended:

- Legal Regulations: It is essential to define the concept of “cosmeceutical” in legal regulations and establish a special oversight mechanism for this category that balances cosmetic and drug regulations. This will both ensure consumer protection and prevent unfair competition in the industry. The development of “quasi‐drug” classifications, similar to those in Japan, could be a model for legally acknowledging products that have therapeutic actions but fall short of qualifying as drugs [46].
- Education and Awareness: Comprehensive media literacy education should be provided to the public, especially social media users, about the risks of aesthetic procedures and realistic expectations. Physicians, being more proactive in the informed consent process, will ensure that patients fully understand the potential risks [47].
- Professional Standards: Medical professional organizations and health authorities should increase interdisciplinary collaboration and establish clear and up‐to‐date standards on which professionals can perform which procedures. These standards should define a clear scope of practice, especially for non‐surgical semi‐invasive procedures, and maintain patient safety at the highest level. *Practitioners accredited by the Joint Council for Cosmetic Practitioners (JCCP) in the United Kingdom provide a valuable model for establishing and enforcing professional standards in non‐surgical cosmetic procedures* [47]. *The JCCP framework demonstrates how a multidisciplinary council, supported by Professional Standards Authority (PSA) accreditation, can set competencies, regulate training providers, and enhance patient safety. However, regulatory approaches vary widely across countries. While the JCCP represents a voluntary, multidisciplinary model, other nations rely primarily on statutory state regulation or professional councils*. These differences are illustrated in Table 3, which provides a cross‐country comparison of regulatory models for non‐surgical cosmetic procedures.

**TABLE 3:** Cross‐country comparison: organizations and regulatory models for non‐surgical cosmetic procedure.^a^

Country	Institution/System	Type of structure	Scope/Authority
United Kingdom	JCCP (Joint Council for Cosmetic Practitioners)	Voluntary council + PSA (Professional Standards Authority) accreditation	Sets training standards, registers practitioners & training providers, issues patient safety guidance
Netherlands	BIG‐register (Beroepen in de Individuele Gezondheidszorg)	Statutory/mandatory state register	Defines exactly which health professionals may perform which procedures
Germany	State health authorities	State regulation	Botulinum toxin/dermal fillers restricted to physicians only
France	Ordre des Médecins (Medical Council)	Professional regulatory body	Non‐surgical invasive procedures limited to doctors only

^a^ Data compiled from the Joint Council for Cosmetic Practitioners (JCCP) [47], the BIG‐register in the Netherlands [48], German state health authorities [49], and the Ordre des Médecins in France [50].

This narrative review has several limitations related to its legal and regulatory focus. The analysis is based on existing laws, regulations, and professional guidelines rather than original clinical data; therefore, it does not quantify patient outcomes, complication rates, or the direct impact of regulatory gaps on patient safety. In addition, legal definitions, enforcement mechanisms, and scope‐of‐practice regulations differ across jurisdictions, limiting the direct generalizability of some comparisons. Furthermore, unsafe or unregulated cosmetic practices that may result in patient harm are likely underreported in the available literature.

Future research should focus on interdisciplinary studies examining the relationship between regulatory deficiencies and patient safety in cosmetic procedures with medical implications. Comparative legal research assessing licensing systems, professional oversight, and enforcement strategies would be valuable. Clinical and medico‐legal studies evaluating adverse events, delayed diagnosis, and complication management in non‐medical cosmetic settings are also needed to support the development of clearer legal boundaries and stronger patient protection.

In conclusion, aesthetic medicine can advance in a safe and sustainable manner not only through technological innovation but also by embracing an approach that institutionalizes interdisciplinary collaboration, clarifies legal frameworks, and prioritizes ethical principles. Such an approach will ensure that patient safety remains paramount while maintaining the professional integrity of medical practice.

## Author Contributions

Eda Kumbasar‐Atay and Kadircan H. Keskinbora contributed to the conception, drafting, and critical revision of the manuscript. Both authors approved the final version.

## Funding

The authors have nothing to report.

## Ethics Statement

The authors have nothing to report.

## Consent

The authors have nothing to report.

## Conflicts of Interest

The authors declare no conflicts of interest.

## Data Availability

The data that support the findings of this study are available on request from the corresponding author. The data are not publicly available due to privacy or ethical restrictions.
